# Preparation of Monoclonal Antibodies against the Viral p54 Protein and a Blocking ELISA for Detection of the Antibody against African Swine Fever Virus

**DOI:** 10.3390/v14112335

**Published:** 2022-10-25

**Authors:** Yanni Gao, Tingting Xia, Juan Bai, Lujie Zhang, Haixue Zheng, Ping Jiang

**Affiliations:** 1 Key Laboratory of Animal Diseases Diagnostic and Immunology, Ministry of Agriculture, MOE Joint International Research Laboratory of Animal Health and Food Safety, College of Veterinary Medicine, Nanjing Agricultural University, Nanjing 210095, China; 2 State Key Laboratory of Veterinary Etiological Biology and World Organisation for Animal Health/National Foot and Mouth Disease Reference Laboratory, Lanzhou Veterinary Research Institute, Chinese Academy of Agricultural Sciences, Lanzhou 730046, China; 3 Jiangsu Co-Innovation Center for the Prevention and Control of Important Animal Infectious Diseases and Zoonoses, Yangzhou University, Yangzhou 225009, China

**Keywords:** African swine fever virus, p54 protein, monoclonal antibodies, blocking ELISA

## Abstract

African swine fever virus (ASFV) causes a highly contagious viral disease in domestic and wild pigs, leading to serious economic losses. As there are no vaccines or drugs available, early accurate diagnosis and eradiation of infected animals are the most important measures for ASFV prevention and control. Therefore, improvement of available diagnostic assays and development of novel effective techniques are required. This study is devoted to generating a new detection platform of blocking monoclonal antibody-based enzyme-linked immunosorbent assay (ELISA) against ASFV p54 protein. Seven monoclonal antibodies against recombinant p54 protein were produced and four epitopes were identified. Three blocking ELISAs were developed with 6A5 and 6F9 mAbs labeled with HRP, respectively, of which the 6A5/6F9-based blocking ELISA displayed the best detection performance, with an AUC of 0.986, sensitivity of 98.36% and specificity of 92.36% in ROC analysis. Moreover, it has an excellent agreement at 96.59% (198/205) when compared to the commercial blocking ELISA (kappa value = 0.920). The method also has high repeatability, with CV <10%, and no cross reaction with the serum antibodies against PRV, PRRSV, CSFV, PCV2 or SVA. This indicates that the 6A5/6F9-based blocking ELISA has high accuracy with good sensitivity and specificity, suitable for viral detection, field surveillance and epidemiological studies.

## 1. Introduction

African swine fever virus (ASFV) causes a highly contagious viral disease in swine, with acute hemorrhagic fever, showing high mortality—approaching 100%—in domestic pigs [1]. It is a large, enveloped virus with icosahedral morphology [2] and belongs to the Asfarviridae family, isolates of which have linear dsDNA genomes of 170–194 kbp, encoding more than 150 polypeptides [3].

To date, African swine fever (ASF) is one of the most devastating diseases threatening pigs and wild suids [4], leading to serious economic and production losses. ASF is endemic in Sub-Saharan Africa [5], but since its introduction to the Caucasus region in 2007, a highly virulent strain of ASFV has continued to circulate and spread into Eastern Europe and Russia, and most recently into Western Europe and Asia [3]. This is of particular concern in the case of China, which produces half of the world’s pig population, where ASF was first reported in 2018 [6].

Currently, there are no safe and effective vaccines or drugs available to prevent and control ASFV outbreaks [7]. ASF control is mainly dependent on early diagnosis and elimination of infected animals [8]. Therefore, highly sensitive and specific diagnostic assays are critical for rapid and early detection of infected pigs. Serological assays are commonly used for diagnosis as they are simple, cost-effective and comparatively safe, with no need for virus isolation or viral genome extraction [9]. The IgG antibody could be detected 7–10 days post ASFV infection and persists for a long time [10]. Since no commercial vaccines against ASF have been developed, the presence of ASFV antibodies indicates current or historic infection [11]. Among different immunoassay approaches, monoclonal antibody (mAb)-based blocking enzyme-linked immunosorbent assays (ELISAs) are highly specific for ASFV antibody detection, reducing the number of false positive tests during surveillance of a negative population [12]. As a result, it is important to develop an accurate mAb-based blocking ELISA for detection of ASFV.

MAbs against ASFV’s major capsid protein p72 and the structural and highly immunogenic protein p30 have been used as targets for detection of ASFV infection in serological assays [11,12]. The type II transmembrane protein p54, which plays a key role in virus morphogenesis and viral infection, was also suggested as an important detection target [13,14]. ASFV infection or p54 replicon inoculation both induced high titers of anti-p54 antibodies [15,16,17,18,19]. Furthermore, antibodies against p54 appear as early as 10 days post infection and persist within the blood for a few weeks [16,17]. Therefore, serological tests against p54 would likely be effective for ASFV detection.

In the present study, a panel of mAbs against *Escherichia coli* (*E. coli*)-expressed p54 recombinant protein was generated and characterized. Due to the high and specific blocking activity of two mAbs, a blocking ELISA based on p54 was developed. The established blocking ELISA was sensitive and specific for ASFV antibody detection, providing a new tool for ASFV surveillance.

## 2. Materials and Methods

### 2.1. Cells, Sera and Animals

SP2/0 myeloma cells were preserved by Key Laboratory of Animal Diseases Diagnostic and Immunology, College of Veterinary Medicine, Nanjing Agricultural University (Nanjing, China). ASFV-positive and -negative sera, and the inactivated ASFV with 4% paraformaldehyde (PFA), were kindly provided by Lanzhou Veterinary Research Institute, Chinese Academy of Agricultural Sciences. BALB/c mice were purchased from Yangzhou University Experimental Animal Center.

### 2.2. Construction of p54 Recombinant DNA

**Figure 1:**
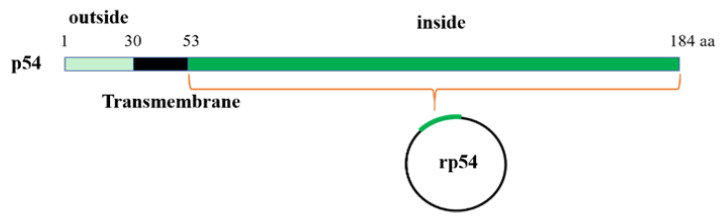
Strategy of the truncated recombinant p54 (rp54) expression.

The pcDNA3.1-p54 plasmid was used for the preparation of partial p54 DNA fragments (excluding the outer- and trans-membrane domains, Figure 1). The primer was designed based on ASFV isolate Pig/HLJ/2018 (accession. No. MK333180.1) [20] and used to amplify the p54 gene with a forward primer, 5′-CGCGGATCCTCTTCAAGAAAGAAAAAAGC-3′, and a reverse primer, 5′-TACCTCGAGCAAGGAGTTTTCTAGGTCTTTATG-3′. The amplicon was cloned into pET-28a with *BamHI* and *XhoI* restriction enzyme sites. The recombinant plasmids were then transformed into Rossetta (DE3) *E. coli* competent cells (TransGen Biotech Co., Ltd., Beijing, China) for overnight incubation at 37 °C in a kanamycin-treated agar plate. The recombinant plasmids were then extracted and checked by restriction enzymes, and positive samples were further confirmed by DNA sequencing (Sangon Biotech Co., Ltd., Shanghai, China).

### 2.3. Expression and Purification of ASFV Recombinant p54 (rp54) Protein

The confirmed rp54 DNA was cultured in Luria–Bertani liquid medium with 100 µg/mL of kanamycin. When the optical density at 600 nm of the culture reached 0.6, protein expression was induced with 0.5 mM isopropyl-β-D-1-thiogalactoside (IPTG) at 37 °C for 6 h. Sodium dodecyl sulfate–polyacrylamide gel electrophoresis (SDS-PAGE) analysis was performed to examine the rp54 expression with cell lysates. For rp54 protein purification, bacterial cells were harvested by centrifugation at 4000 rpm for 15 min, resuspended in cold PBS and lysed by sonication (300 W, 6 s bursts with 6 s pauses) for 30 min on ice. Supernatants were collected from the cell lysates after centrifugation at 12,000 rpm for 30 min. A 0.22 µm filter was used to filter the supernatant before rp54 protein was purified through a Ni-NTA resin-based column. The purified rp54 protein was analyzed and verified by SDS-PAGE and western blot with anti-His mAbs (Proteintech Group, Inc., Rosemont, IL, USA) and ASFV-positive serum as primary antibodies.

### 2.4. Rp54 mAbs Production

Hybridoma technology was applied for production of anti-rp54 mAbs, as previously described [21]. In brief, antigen was prepared with equal volume of purified rp54 protein and incomplete Freund’s adjuvant (Sigma-Aldrich (Shanghai) Trading Co. Ltd., Shanghai, China). Female mice (BALB/c), 6–8 weeks of age, were intraperitoneally injected with 100 µg/mouse of the prepared antigen. The mice were immunized three times with 3-week intervals between each immunization. At 10 days post the final immunization, the immunized mice were euthanized. Splenocytes were collected for fusion with SP2/0 myeloma cells, after which the fused cells were cultured with HAT selection media in 96-well plates. Cell supernatants were collected at 7 days post cell fusion and screened for anti-rp54 antibodies by indirect ELISA coated with rp54 protein. Hybridoma clones that secreted rp54-specific antibodies were then subcloned into single-cell clones by three rounds of limiting dilution. Ascites was prepared from 10-week-old female BALB/c mice inoculated with hybridomas.

### 2.5. Indirect ELISA

Purified rp54 protein was diluted in carbonated coating buffer (pH = 9.6) at 2 µg/mL and used to coat flat-bottom polystyrene plates (100 µL/well) by overnight incubation at 4 °C. The plates were washed with PBST (0.05% Tween in PBS, *v*/*v*) three times before being blocked with 5% skimmed milk in PBS for 2 h at 37 °C. Then, the plates were washed another three times, and 100 µL hybridoma supernatants were loaded; positive sera from rp54 recombinant protein-immunized mice and negative sera from unimmunized mice (1:1000 dilution) were used as controls. After 60 min incubation at 37 °C and another washing step, horseradish peroxidase (HRP)-conjugated goat anti-mouse IgG (Beyotime Biotechnology Co., Ltd., Shanghai, China) (1:1000 dilution) was added for 45 min incubation at 37 °C. Following washing three times, a chromogenic substrate solution (TMB) (Beyotime Biotechnology Co., Ltd., Shanghai, China) was added and incubated for 10 min before being stopped with 2M H_2_SO_4_. The plates were read at 450 nm.

### 2.6. Western Blot Analysis

Western blot was performed to detect the reactivity of the monoclonal antibodies against rp54 recombinant protein. Purified His-tagged rp54 protein was electrophoresed in a 12.5% SDS-PAGE, before being transferred onto nitrocellulose filter membrane. Then, the membrane was blocked with 5% skimmed milk by 2 h incubation at room temperature. Primary antibody incubation was performed with seven anti-rp54 monoclonal antibodies (hybridoma supernatant) and anti-His monoclonal antibodies (1:5000 dilution), respectively, for 2 h at room temperature. Washing five times with PBST was performed before HRP-labelled goat anti-mouse IgG was added for 1 h incubation at room temperature. After a final wash step, the membrane was screened by a digital imaging system.

### 2.7. Indirect Immunofluorescent Assay

Indirect Immunofluorescent Assay (IFA) was conducted on 4% PFA-treated ASFV-infected PAMs (a kind gift provided by Lanzhou Veterinary Research Institute). PAMs were collected from 4-week-old pigs and seeded onto 96-well plates, incubated in RPMI 1640 medium (Gibco, Thermo Scientific, Waltham, MA, USA) with 10% FBS (Gibco, Thermo Scientific, Waltham, MA, USA) at 37 °C with 5% CO_2_. PAMs were infected with ASFV (MOI = 0.1) for 48 h before the cells were fixed with 4% PFA in PBS for 30 min at room temperature and then stored at 4 °C. Experiments with live ASFV were performed in the Biosafety Level 3 Laboratory of Lanzhou Veterinary Research Institute. The fixed cells were incubated with anti-rp54 mAb before incubation with Coralite488-conjugated Affinipure Goat Anti-Mouse IgG(H+L) (Proteintech Group, Inc., Rosemont, IL, USA), and the plates were examined by Zeiss Axio Observer.

### 2.8. Antigen Epitope Analysis 

To map the antigen epitopes that the seven anti-rp54 mAbs recognize, 11 truncated rp54 fragments were constructed in pET-32a and expressed in *E. coli*. The reactivities of the seven mAbs against these truncated proteins were detected by western blot, as described above. Sequence conservation of the recognized epitopes among different ASFV genotypes was analyzed by the clustalW multiple method, using BioEdit 7.0 software (http://www.mbio.ncsu.edu/BioEdit/page2.html, accessed on 12 December 2021). The reference sequences were obtained from the Genbank database.

### 2.9. Assessment of the Potential Use of the mAbs for Blocking ELISA

To evaluate the potential use of these anti-rp54 mAbs as a diagnostic reagent for ASFV antibody detection, blocking ELISA based on each mAbs was investigated. Strong positive (S1), medium positive (S2), weak positive (S3) and negative (N1) sera were utilized to determine which mAbs were appropriate for application in blocking ELISA. Each sample was tested with the blocking ELISA at a dilution of 1:2, and the PI value of each sample was calculated.

### 2.10. Development of MAb-Based Blocking ELISA for Detection of African Swine Fever Antibody 

The purified rp54 mAbs were labeled with HRP (Biodragon Biotechnology Co., Ltd., Beijing, China) to establish blocking ELISA for detection of ASFV antibodies. Purified rp54 protein was diluted in carbonated coating buffer (pH = 9.6) and coated on flat-bottom polystyrene plates (100 µL/well) for overnight incubation at 4℃. The plate was washed three times with PBST and blocked with 1% BSA (Sigma, St. Louis, MO, USA) in PBS for 1h–3 h at 37 °C. After a wash step, 100 µL of testing serum and controls were added for 1 h incubation at 37 °C. All samples were diluted at 1:1 in dilution buffer (0.01% Tween 20 in PBS). Another wash step was performed before the plates were incubated with 100 µL of HRP-conjugated anti-rp54 mAb at 37 °C for another 30–45 min. Then, the plates were washed three times with PBST, followed by reaction with TMB for 15–20 min, before being stopped with Stop Solution for TMB Substrate (Beyotime Biotechnology Co., Ltd., Shanghai, China). The plates were read at 450 nm. Raw data were collected and the percent of inhibition (PI value) of each test sample was calculated according to PI (%) = [(OD_450_ value of negative controls − OD_450_ value of sample)/OD_450_ value of negative controls] × 100% [22]. More details about the blocking ELISA are listed in Table 1.

### 2.11. Determination of Cut-Off Value, Diagnostic Sensitivity and Diagnostic Specificity 

Cut-off values with associated diagnostic sensitivity and specificity were determined with serum samples from ASFV-positive and -negative individual pigs by blocking ELISA. A commercial blocking ELISA kit (ID Screen^®^ African Swine Fever Competition ELISA, IDVET, Grabels, France) was used as a standard evaluating method. Receiver operating characteristic (ROC) analysis, degree of agreement (kappa value) and the sensitivity and specificity of the established ELISA were analyzed by SPSS software for windows, version 25.0 (IBM, Armonk, NY, USA).

### 2.12. Analytical Specificity and Analytical Sensitivity of Blocking ELISA 

To evaluate the analytical specificity, five polyclonal anti-sera against other swine viruses (PRRSV, PRV, CSFV, SVA and PCV2) were detected by the developed blocking ELISA.

Analytical sensitivity of blocking ELISA was determined by two-fold serial dilution of 5 positive sera and 3 negative sera in the range of 1:2–1:128.

### 2.13. Assessment of Blocking ELISA Repeatability and Reproducibility

To assess the repeatability and reproducibility of the developed blocking ELISA, 5 positive control and 5 negative control samples were tested using the ELISA on one plate in one run or on three plates in three independent runs. Each serum was tested in triplicate. SPSS software (Windows, version 25.0.) was used to calculate the means, standard deviations and percent coefficient of variation (% CV). 

### 2.14. Detection of ASFV Antibodies in Pig Sera

To detect ASFV antibodies in field pig sera, a total of 330 field serum samples collected from different regions of China between June 2021 and January 2022 with unknown exposure to ASFV were selected and tested by the newly developed blocking ELISA. 

## 3. Results

### 3.1. Antigen Preparation

**Figure 2:**
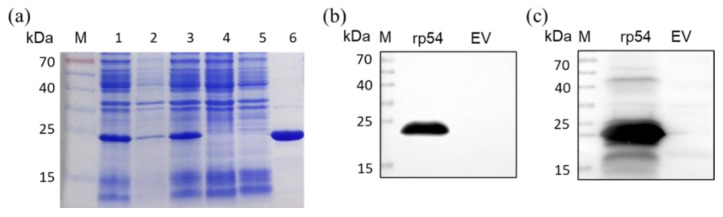
Analysis of rp54 protein. (**a**). SDS-PAGE analysis of His-tagged rp54 protein expression, followed by Coomassie brilliant blue stain. M is protein ladder, lane 1 was supernatants of bacterial cell lysates after induction with IPTG, lane 2 was precipitates of bacterial cell lysates after induction with IPTG, lane 3 was whole bacterial cell lysates after induction with IPTG, lane 4 was whole bacterial cell lysates with no induction with IPTG, lane 5 was negative control with pET-28a vector, lane 6 was the purified rp54 protein. (**b**). Western blot analysis of rp54 protein with anti-His tag antibody. (**c**). Western blot analysis of rp54 protein with ASFV-positive serum.

The recombinant pcDNA3.1-p54 plasmid was cloned and transformed into *Rossetta* to produce. The rp54 protein (20 kDa) was expressed in a high yield in the supernatant of the cell lysates (Figure 2a). The fusion protein was purified using the Ni-NTA resin-based column, and confirmed with an anti-His mAb and ASFV-positive serum, respectively, by western blot (Figure 2b,c).

### 3.2. Generation of MAbs against ASFV rp54

**Figure 3:**
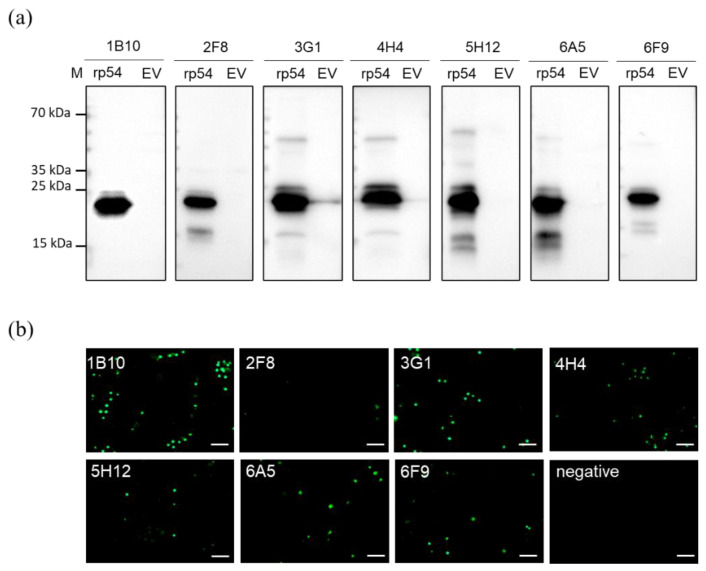
The reactivity of monoclonal antibodies. (**a**). Western blot analysis of anti-rp54 antibodies. The seven anti-rp54 monoclonal antibodies specifically reacted with recombinant protein at 20 kDa. (**b**). IFA performed on ASFV-infected PAMs. Cells were incubated with rp54-specific mAbs annotated on the top left of each panel and stained with Coralite488-conjugated Affinipure Goat Anti-Mouse IgG(H+L). Scale bars, 20 μm.

Seven positive clones specific to rp54 protein designated as 1B10, 2F8, 3G1, 4H4, 5H12, 6A5 and 6F9 were obtained and subcloned three times by limiting dilution. The seven mAbs were further verified by western blot (Figure 3a) and IFA using PAMs infected with ASFV (Figure 3b). Monoclonal antibodies isotypes were characterized using a Mouse Ig Isotyping Kit (Proteintech Group, Inc., Rosemont, IL, USA). 2F8, 3G1, 4H4 and 5H12 were found to be IgG1 with kappa light chain and 1B10, 6A5 and 6F9 were IgG2a with kappa light chain (Table 2).

### 3.3. Identification and Conservation Analysis of the rp54 Epitopes

**Figure 4:**
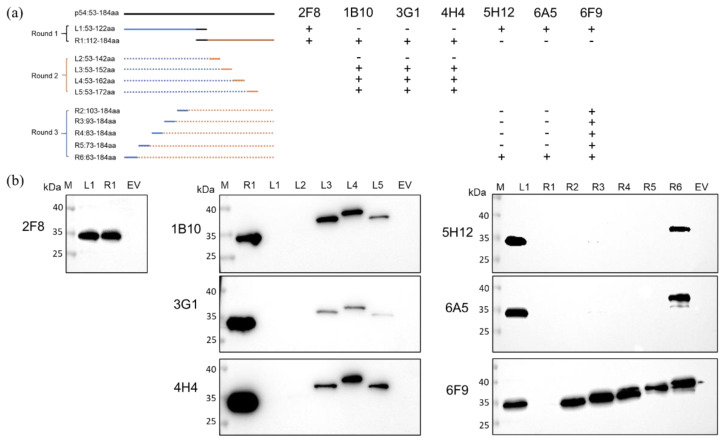
Antigen epitope analysis. (**a**). Strategy for the identification of the epitopes within rp54 protein and reactivity between the truncated rp54 protein fragments and the seven mAbs. +: interaction; −: no interaction. (**b**). Western blot analysis of the reactivity of the truncated rp54 protein fragments and the seven mAbs.

**Figure 5:**
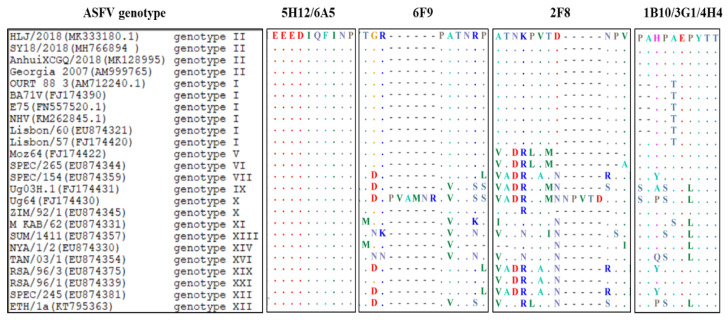
Sequence conservation analysis of the identified epitopes among different ASFV genotypes.

To determine the epitopes recognized by the seven mAbs, 11 overlapping peptide fragments spanning the rp54 protein were expressed and subjected to western blot analysis to test their reactivity with the seven mAbs within three rounds (Figure 4a). As shown in Figure 4b, 2F8 mAb bound to both L1 and R1, indicating that its recognized epitope was located within ^112^ATNKPVTDNPV^122^ (2F8-Epi); 1B10, 3G1 and 4H4 bound to R1, L3, L4 and L5, but not L1 and L2, indicating their recognized epitopes were located within ^143^PAHPAEPYTT^152^ (1B10, 3G1, 4H4-Epi); 5H12 and 6A5 bound to L1 and R6, but not R1-R5, indicating that their recognized epitopes were located within ^63^EEEDIQFINP^72^ (5H12, 6A5-Epi); 6F9 recognized L1 and R2-R6, but not R1, indicating that its targeting epitope was located within ^103^TGRPATNRP^111^ (6F9-Epi). Among these, ^143^PAHPAEPYTT^152^ is a novel epitope of p54 protein. Further conservation analysis of the recognized epitopes among different ASFV genotypes by BioEdit 7.0 software demonstrated that the 5H12/6A5-Epi was highly conserved among all ASFV genotypes analyzed; 2F8-Epi was conserved among ASFV genotype I and II; 6F9-Epi were conserved among ASFV genotype I, II, V and VI; and 1B10, 3G1 and 4H4-Epi was conserved in ASFV genotype II (Figure 5).

### 3.4. Assessment of Potential Use of the Rp54 MAbs for Blocking ELISA

**Figure 6:**
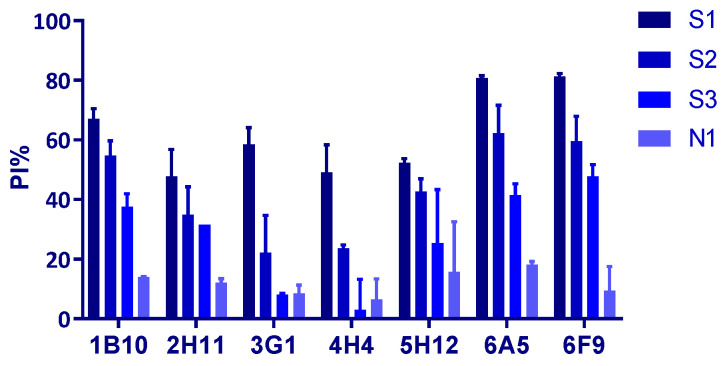
Assessment of rp54 mAbs for application in blocking ELISA against ASFV antibodies. The percent of inhibition of different mAbs against strong-, medium-, weak-positive (S1, S2 and S3, respectively) and negative serum (N1) samples were determined.

The PI values of the sera in blocking ELISA with these seven anti-rp54 mAbs were analyzed to determine which mAbs were appropriate for application in blocking ELISA. As shown in Figure 6, 6A5 and 6F9 showed a greater blocking capacity compared to other mAbs.

### 3.5. Standardization and Determining the Cut-Off Value for Blocking ELISA

**Figure 7:**
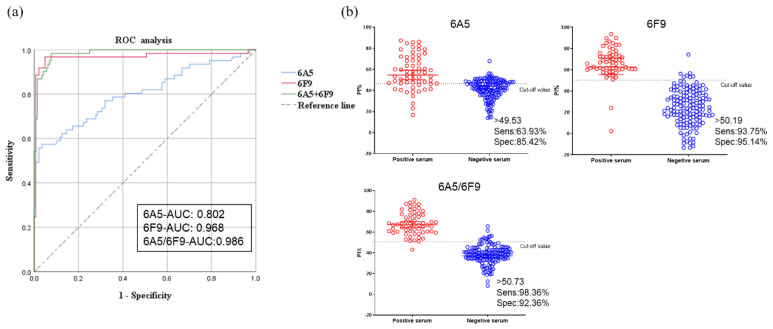
Serum sample analysis by 6A5, 6F9 and 6A5/6F9-based blocking ELISAs. 61 ASFV-positive serum samples and 144 ASFV-negative serum samples were analyzed by the three blocking ELISAs. (**a**). ROC analysis of the three blocking ELISAs. The AUC was 0.802, 0.968 and 0.986 for 6A5, 6F9 and 6A5/6F9-based blocking ELISAs, respectively. (**b**). PI values of each pig serum sample, labelled with indicated cut-off values, sensitivity and specificity.

Since 6A5 and 6F9 exhibited a good blocking capacity and were highly conservative among different ASFV genotypes, they were used to develop blocking ELISAs with a single or combination of these two mAbs (6A5/6F9), respectively. After protocol optimization for blocking ELISA, a total of 205 pig serum samples (61 ASFV-positive samples and 144 ASFV-negative samples) were used to evaluate the developed blocking ELISAs. The serum samples were identified as ASFV-positive or -negative according to their known origin and confirmed by a commercial ASFV antibody detection kit (ID Screen^®^ African Swine Fever Competition ELISA, IDVET, France)^®^. Each sample was tested by the developed blocking ELISAs in duplicates to calculate the percent of inhibition value. ROC curve statistical analysis was performed and the cut-off value, as well as the corresponding diagnostic sensitivity and specificity, were determined (Figure 7). An interactive dot plot diagram with the blocking value of these samples is shown in Figure 7b. An area under the curve (AUC) of 1 represents a perfect test, and an AUC over 0.9 indicates the assay has high accuracy. According to the ROC analysis, the AUC was 0.802 (95% confidence interval: 0.872 to 0.876), based on 6A5, 0.968 (95% confidence interval: 0.933 to 1.000), based on 6F9, and 0.986 (95% confidence interval: 0.973 to 0.998), based on 6A5/6F9. Moreover, for blocking ELISA based on 6A5, a diagnostic sensitivity of 63.93% and a specificity of 85.42% were achieved when the cut-off value was set to 49.53%; for blocking ELISA based on 6F9, a diagnostic sensitivity of 93.75% and a specificity of 95.14% were achieved when the cut-off value was set to 50.19%; and for blocking ELISA based on 6A5/6F9, a diagnostic sensitivity of 98.36% and a specificity of 92.36% were achieved when the cut-off value was set to 50.73%. The blocking ELISA based on 6A5/6F9 was the most accurate and robust candidate assay.

### 3.6. Analytical Specificity and Analytical Sensitivity of Blocking ELISA 

**Figure 8:**
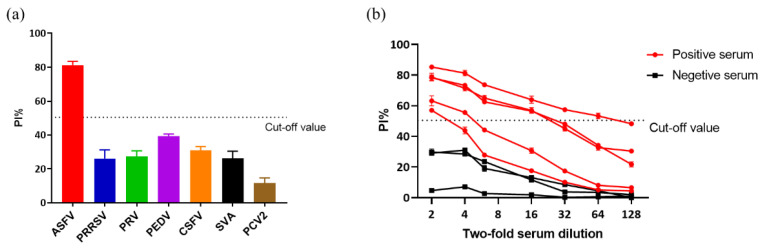
Specificity and analytical sensitivity assay. (**a**). Percent of inhibition of the polyclonal anti-sera against various porcine viruses analyzed by 6A5/6F9-based blocking ELISA. Only ASFV-positive pig serum showed a higher PI value than the cut-off value. (**b**). Two-fold serially diluted ASFV-positive sera (Red) and ASFV-negative sera (Black) ranging from 1:2 to 1:128 were detected by the blocking ELISA. Cut-off value was marked with a dashed line.

To evaluate the specificity of the developed 6A5/6F9-based blocking ELISA, six polyclonal anti-sera against other swine viruses (PRRSV, PRV, PEDV, CSFV, SVA and PCV2) were detected by the assay. As shown in Figure 8a, all sera against other swine viruses were tested as negative by blocking ELISA with a much lower blocking value compared to the cut-off value. Therefore, the non-specific positive swine serum could clearly be distinguished from the ASFV-positive serum, indicating that the established blocking ELISA was highly specific for ASFV detection. 

The analytical sensitivity of the 6A5/6F9-based blocking ELISA was evaluated using five positive sera and three negative sera against ASFV with two-fold dilutions. As shown in Figure 8b, the highest dilution at 1:64 of the detected ASFV-positive serum sample produced a positive test result in the 6A5/6F9-based blocking ELISA, indicating that the assay showed a good analytical sensitivity.

### 3.7. Assessment of Blocking ELISA Repeatability and Reproductivity

To evaluate the repeatability of the 6A5/6F9-based blocking ELISA, 10 serum samples (five positive samples and five negative samples) were tested with this method. The coefficient of variation (CV) was calculated to determine the intra- and inter- assay variation. The assay is considered to have adequate repeatability when the intra-assay CV is <10%, and adequate reproductivity when the inter-assay CV is <10%. In this study, as shown in Table 3, the intra-assay CV of the PI ranged from 0.17% to 9.92%, while the inter-assay CV of the PI ranged from 1.54% to 9.50%, indicating a good repeatability and reproducibility of the 6A5/6F9-based blocking ELISA.

### 3.8. Presence of ASFV Antibodies in Pig Sera

A total of 28 out of the 330 field sera (28/330) of pigs collected from Henan, Jiangsu, Shandong and Zhejiang provinces of China, from June 2021 to January 2022, with unknown exposure to ASFV, were found to be positive when tested by the established 6A5/6F9-based blocking ELISA, of which 20 were positive in sera from Shandong (20/100), and four sera from Henan (4/92) and Jiangsu (4/106), respectively (Table 4).

## 4. Discussion

African swine fever (ASF) is a highly contagious viral disease of domestic and wild pigs, which is responsible for serious economic and production losses [23]. Its causative agent, ASFV, belongs to the *Asfarviridae* family in the nucleocytoplasmic large DNA virus superfamily [24]. In addition, it is divided into 24 genotypes according to its B646L gene sequences [25], of which the genotype II is the main epidemic strain in China [26]. Currently, there are no approved vaccines or antiviral drugs against ASF [7]. During outbreaks of ASF in affected countries, classic sanitary measures, including early detection and humane killing of the infected animals, are the most effective [27]. Therefore, screening of the infected animals as early as possible is of great significance for ASF prevention and control. As clinical symptoms of ASFV-infected animals vary considerably from acute forms with a mortality rate of 90–100% in domestic pigs and wild boar to subclinical infections in bushpigs and warthogs [28], and the clinical symptoms between ASF and classical swine fever infection demonstrate no significant differences [29], more accurate laboratory detection methods are required to determine the infection of animals. 

Antibody detection by serological assays is widely used for surveillance of ASFV infection, as antibodies against ASFV appear soon after infection and persist for a long time [30]. ELISA is a common tool for serological surveillance. In blocking ELISA, virus-specific antibodies in samples bind the antigens and block the binding of a mAb to the antigens. It has been wildly applied for serological diagnosis of various diseases with high accuracy and specificity [31,32].

In the present study, we first produced seven mAbs against the *E. coli*-expressed rp54 protein. Analysis of those mAbs with ELISA, western blot and IFA showed that all seven monoclonal antibodies were able to recognize the immunizing rp54 antigen. The epitopes within rp54 that were recognized by these seven mAbs were identified and their sequence conservation among different ASFV genotypes was analyzed. 6A5-Epi demonstrated an absolute conservation among the 14 ASFV genotypes analyzed, and 6F9-Epi was highly conservative in ASFV genotypes I, II, V and VI. Moreover, assays examining the competing abilities of the seven mAbs demonstrated that 6A5 and 6F9 displayed comparatively high PI values, indicating that they were appropriate for application in blocking ELISA for ASFV antibody detection. Therefore, three blocking ELISAs based on 6A5, 6F9 and 6A5/6F9 were generated, respectively. Accuracy, sensitivity and specificity of these three blocking ELISAs were analyzed with 205 pig sera and a commercially available blocking ELISA kit (ID Screen^®^ African Swine Fever Competition ELISA, IDVET, France) for ASFV antibody detection was taken as a standard evaluating method. A receiver operating characteristic (ROC) analysis was performed with the PI values of 205 pig serum samples. The results show that the 6A5/6F9-based blocking ELISA displayed the best performance, with an AUC of 0.986, indicating the very high accuracy of this method. When the cut-off value was set to 50.73%, an optimal balance of sensitivity (98.36%) and specificity (92.36%) was obtained. The high sensitivity of the assay makes it an effective tool for screening ASFV in the field. Moreover, the detection results achieved from this 6A5/6F9-based blocking ELISA show an excellent agreement (kappa value = 0.920) with those obtained from the commercially available blocking ELISA kit (ID Screen^®^ African Swine Fever Competition ELISA, IDVET, France), indicating a good concordance between these two methods. The specificity test manifested that the 6A5/6F9-based blocking ELISA definitely distinguished ASF from other common pig diseases, and the repeatability assay confirmed the reproducibility of this method. The analytical sensitivity of the 6A5/6F9-based blocking ELISA was detected by using a dilution of 1:2 to 1:64 for different ASFV-positive serum samples, as per the previous description using the recombinant p30 protein of ASFV [10]. The results show that it had good analytical sensitivity. Furthermore, this assay was used to detect ASFV antibodies in a total of 330 field serum samples collected from different regions of China. In addition, 28 out of the 330 field sera were found to be ASFV-positive, which is consistent with the results of another report by an ecological niche model based on ensemble algorithms [33]. 

So far, the commercially available blocking ELISA kits for ASFV antibody detection are limited to detection against anti-p30 and -p72 antibodies. The 6A5/6F9-based blocking ELISA generated in this study, with high accuracy, sensitivity and specificity, provides a new method targeting p54 protein for ASFV detection. The high sequence conservations of the 6A5 and 6F9 mAbs-recognized epitopes within p54 protein among various ASFV genotypes suggest the broad detection spectrum of this 6A5/6F9-based blocking ELISA. Although further validation of this method with a large scale of samples is still needed, the current work provides a new platform for ASFV antibody detection by blocking ELISA.

## Figures and Tables

**Table 1:** The optimization of react condition of ASFV p54 protein blocking enzyme-linked immunosorbent assay (ELISA).

Blocking ELISA Method	Optimized Conditions	Antigen Coating	Blocking Conditions	Serum to be Tested	HRP Labeled Antibody	TMB Reaction Time
**6A5**	Concentration/dilution	0.25 μg/mL	1% BSA	1:1	1:3000 (0.77 μg/mL)	
	Reaction conditions	4 °C 12 h	37 °C 1 h	37 °C 1 h	37 °C 30 min	37 °C 20 min
**6F9**	Concentration/dilution	0.25 μg/mL	1% BSA	1:1	1:2000 (0.95 μg/mL)	
	Reaction conditions	4 °C 12 h	37 °C 1 h	37 °C 1 h	37 °C 40 min	37 °C 15 min
**6A5+6F9**	Concentration/dilution	0.5 μg/mL	1% BSA	1:1	6A5—1:3000 mix with 6F9—1:2000	
	Reaction conditions	4 °C 12 h	37 °C 1 h	37 °C 1 h	37 °C 30 min	37 °C 20 min

**Table 2:** Identification of subclasses of anti-p54 monoclonal antibodies.

	Monoclonal Antibodies						
	1B10	2F8	3G1	4H4	5H12	6A5	6F9
Ig subclass	IgG2a	IgG1	IgG1	IgG1	IgG1	IgG2a	IgG2a
Light chain type	κ	κ	κ	κ	κ	κ	κ

**Table 3:** Intra- and inter-assay repeatability of the 6A5/6F9-based blocking ELISA.

Samples	Intra-Assay			Inter-Assay		
	Mean PI	SD	CV	Mean PI	SD	CV
Positive1	71.13%	0.001	0.17%	72.36%	0.011	1.54%
Positive2	52.51%	0.008	1.60%	52.65%	0.013	2.50%
Positive3	51.40%	0.004	0.78%	52.11%	0.008	1.56%
Positive4	59.42%	0.036	6.04%	60.67%	0.027	4.48%
Positive5	65.74%	0.034	5.18%	68.28%	0.024	3.54%
Negetive1	21.34%	0.020	9.46%	18.69%	0.004	2.20%
Negetive2	17.13%	0.005	2.85%	16.45%	0.014	8.67%
Negetive3	22.33%	0.013	5.90%	20.47%	0.019	9.50%
Negetive4	16.02%	0.016	9.92%	13.86%	0.008	5.44%
Negetive5	22.07%	0.018	8.03%	24.35%	0.016	6.75%

**Table 4:** Detection of anti-ASFV antibodies in pig serum samples from different provinces.

Province	Serum Samples	Positive Samples	Negative Samples	Positive Rate%
Henan	92	4	88	4.3
Zhejiang	32	0	32	0.0
Jiangsu	106	4	102	3.8
Shandong	100	20	80	20.0
Total	330	28	302	8.5
